# Targeting Epicardial/Pericardial Adipose Tissue in Cardiovascular Diseases: A Novel Therapeutic Strategy

**DOI:** 10.31083/RCM26128

**Published:** 2025-03-13

**Authors:** Yue Ding, Fang Lin, Zhongmin Liu, Xiaohui Zhou, Xiaoting Liang

**Affiliations:** ^1^Department of Organ Transplantation, Changzheng Hospital, Second Military Medical University, 200003 Shanghai, China; ^2^Shanghai Heart Failure Research Center, Shanghai East Hospital, Tongji University School of Medicine, 200120 Shanghai, China; ^3^Translational Medical Center for Stem Cell Therapy & Institute for Regenerative Medicine, Shanghai East Hospital, Tongji University School of Medicine, 200120 Shanghai, China

**Keywords:** epicardial adipose tissue, pericardial adipose tissue, cardiovascular diseases

## Abstract

Cardiovascular diseases (CVDs) remain a global health concern, prompting ongoing research into novel contributors to their pathogenesis. Due to the proximity of the coronary arteries and the myocardium in epicardial adipose tissue (EAT) and pericardial adipose tissue (PAT), these tissues have emerged as key areas of interest for their potential influence on cardiac function and vascular health. This review synthesizes current research on the physiological and biological characteristics of EAT and PAT, exploring their composition and clinical measurement approaches. The roles of EAT and PAT in coronary artery disease (CAD), atrial fibrillation, and heart failure are discussed, and the contributions of EAT and PAT to these cardiovascular conditions are highlighted alongside their potential as therapeutic targets.

## 1. Introduction

Cardiovascular diseases (CVDs) continue to be the leading cause of morbidity and mortality globally, accounting for a significant burden on healthcare systems. Even with advancements in treatments, a pressing need remains to deepen our understanding of the complex mechanisms driving and promoting CVDs. Ongoing research is focused on identifying novel factors contributing to the onset and progression of CVDs, which can lead to improved therapeutic strategies. Among these emerging factors, epicardial adipose tissue (EAT) and pericardial adipose tissue (PAT) have garnered increasing attention for their potential roles in CVD development.

EAT and PAT are fat depots around the heart, but their anatomical positions and biological functions differ. EAT is situated between the heart muscle (myocardium) and the visceral layer of the pericardium, sitting directly next to the coronary arteries and cardiac muscle, whereas PAT lies outside the visceral pericardium, within the parietal pericardium [1]. The proximity of EAT to the crucial cardiac structures, including the coronary arteries, suggests that it could play a direct role in modulating heart function and contribute toward disease pathology. EAT has been reported to exert both local and systemic effects on the heart [2]. Unlike subcutaneous or visceral fat, EAT has no fascial layer separating it from the myocardium; thus, paracrine and vasocrine interactions may occur that influence the onset of coronary artery disease (CAD), atrial fibrillation (AF), and heart failure. Under normal conditions, EAT protects the adjacent myocardium through its brown fat-like thermogenic function. However, these brown fat characteristics gradually diminish in disease states, reducing its thermogenic capacity and protective effects [3, 4, 5]. Instead, EAT begins to secrete proinflammatory and profibrotic cytokines that harm the myocardium by promoting inflammation and fibrosis, thus contributing to and accelerating the progression of CVDs [6, 7].

Building on the role of EAT, the PAT, although more anatomically distant from the myocardium, also contribute significantly to cardiovascular pathology. While the proximity of the EAT promotes its direct interaction with cardiac tissues, PAT influences heart function primarily through systemic mechanisms. Research suggests that PAT volume is linked to higher cardiometabolic indices and subclinical left ventricular deterioration, regardless of general and abdominal obesity [8]. Further, both EAT and PAT have been implicated in adverse cardiac remodeling, particularly in heart failure patients, where the accumulation of these fat depots can exacerbate disease progression. Hence, understanding the distinct yet interconnected roles of these adipose tissues enhances our comprehension of CVDs and underscores the importance of targeting both EAT and PAT in future therapeutic strategies.

To fully explore the potential of targeting EAT and PAT in CVDs, this review aims to consolidate current knowledge on how these fat tissues influence heart health. This review will delve into the mechanisms through which EAT and PAT contribute to CVD pathogenesis, including their roles in CAD, AF, and heart failure. Additionally, we will discuss emerging evidence on the protective functions of EAT and PAT and consider how modifying these tissues could offer new therapeutic avenues. By examining both the harmful and beneficial aspects of EAT and PAT, this review seeks to provide a comprehensive understanding that could inform future strategies for preventing and treating cardiovascular diseases.

## 2. Anatomical and Biological Characteristics of EAT and PAT

**Fig. 1.**
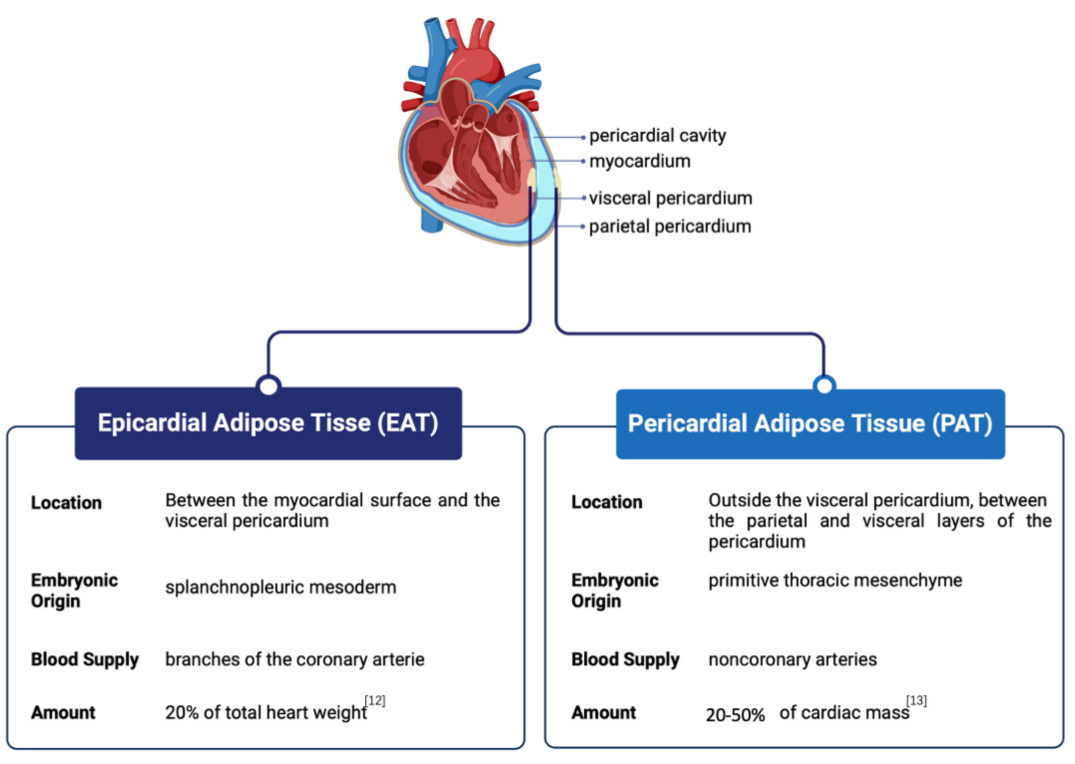
**Anatomical position and comparison of epicardial adipose tissue (EAT) and pericardial adipose tissue (PAT)**. EAT is situated between the myocardial surface and the visceral pericardium, while PAT is located outside the visceral pericardium, between the parietal and visceral layers of the pericardium.

The terms EAT, PAT, and paracardial fat are often used interchangeably in the literature and are collectively referred to as cardiac ectopic fat or cardiac adipose tissue. However, there are two dominating theories regarding their distinction. The most widely accepted view suggests that EAT and PAT are distinct types of adipose tissue, differing in their embryological origins, physiological locations, and functional characteristics. EAT originates from the splanchnopleuric mesoderm [9], whereas PAT stems from the primitive thoracic mesenchyme [10]. Anatomically, EAT is the visceral fat located immediately adjacent to the outer surface of the heart, separated only by the thin epicardium, and blood is supplied from branches of the coronary arteries. EAT covers approximately 80% of the surface of the heart and constitutes approximately 20% of the total weight of the heart in humans and large mammals [11, 12], making it anatomically and functionally contiguous with the myocardium. Comparatively, PAT is positioned within the pericardial sac surrounding the heart, between the parietal and visceral layers of the pericardium, and receives its blood supply from noncoronary arteries. EAT produces and releases numerous bioactive molecules, such as adipokines, cytokines, and inflammatory markers, which exert local effects on the adjacent myocardium, affecting its function and potentially contributing to the development of CVDs. PAT also secretes bioactive molecules, but its distance from the heart muscle may result in a less direct impact on cardiac function than EAT (Fig. 1, Ref. [12, 13]). A less conventional perspective contends that the PAT is composed of two strata: the visceral stratum situated between the myocardium and visceral pericardium, known as the EAT, and the parietal stratum located external to the parietal pericardium, recognized as the paracardial fat layer [14]. Despite the differing viewpoints and approaches to categorization, the clear anatomical distinctions between EAT and PAT and their distinct interactions with the heart underscore the relevance of conducting separate studies on each when their respective contributions to cardiovascular health are examined. Nonetheless, there is a common inclination to confuse the conceptual differences between PAT and EAT, a confusion seen in both humans and rodents regarding cardiac fat terminology. Notably, while EAT is present in humans and other larger mammals, it is either minimally present or entirely absent in experimental animals such as rats or mice [10]. Hence, studies on ‘EAT’ in mice or rats appear to refer to the fat around the heart and not that connected to it. Remarkably, evidence indicates that mouse PAT has features linked to visceral fat precursors, elevated expression of visceral fat-associated genes, and smaller adipocyte size [15, 16]; meanwhile, mice have several pores within their pericardium, facilitating a direct pathway for products originating from the surrounding adipose tissue to reach the myocardium [17]. These findings indicate that mouse PAT could be a visceral fat store potentially relevant to the human concept of EAT. However, no direct evidence currently links rodent PAT to human EAT. This gap further contributes to the disconnect between animal and human studies, limiting our understanding of the functional roles of EAT and PAT across species. Moreover, the question of how to bridge the gap between studying PAT in animals and EAT in humans remains a contentious issue, and resolving this could unlock further insights into the roles these fat depots play in cardiovascular pathology.

## 3. Composition of EAT and PAT

EAT is similar to other visceral fat depots, primarily consisting of adipocytes that store energy as triglycerides. EAT also contains stromal vascular fraction cells such as preadipocytes, fibroblasts, vascular endothelial cells, and immune cells [18]. EAT expands mainly through hyperplasia (increased cell number), as indicated by higher adipocyte density and smaller cell size compared to subcutaneous adipose tissue (SAT) [19, 20]. Additionally, EAT exhibits beige fat-like characteristics, such as a twofold increase in metabolic activity due to enhanced lipolysis and the release of free fatty acids. This increased metabolic activity is supported by a fivefold greater expression of uncoupling protein-1 (UCP-1), which is essential for energy production in brown fat and is absent in other fat tissue types [21]. Furthermore, compared with other adipose tissues, EAT has an improved ability to release free fatty acids into the bloodstream while reducing glucose consumption [22].

Similar to EAT, PAT comprises adipocytes, which store energy as triglycerides, and stromal vascular fraction cells, including preadipocytes, fibroblasts, vascular endothelial cells, and immune cells [23]. However, due to the relatively limited clinical research on PAT compared to EAT and the minimal presence of PAT in commonly used experimental animals (with a typical yield of around 0.005 g per mouse under normal conditions, according to unpublished data from the authors), academic research focusing on PAT also remains scarce.

The amount and distribution of EAT and PAT can vary between individuals and may be affected by factors such as age, sex, ethnicity, and obesity. For example, the average volume of PAT in the Framingham offspring cohort was 137 ± 54 cm^3^ in men and 108 ± 41 cm^3^ in women [24]. Additionally, individuals with a higher body mass index (BMI, >27 kg/m^2^) had more than twice the volume of EAT than those with a BMI below 27 kg/m^2^ (155 ± 15 cm^3^ vs. 67 ± 12 cm^3^) [25]. The unique anatomical positions, metabolic functions, and secretion characteristics of EAT and PAT are closely linked with the development and progression of various CVD types. The following sections explore the complex interactions between EAT/PAT and CVDs, focusing on the mechanisms involved and highlighting recent research advancements in this area.

## 4. Clinical Approaches for EAT/PAT Measurements

Various methods are used to measure EAT and PAT; however, due to the variability in their volumes, no established consensus exists on what constitutes a normal upper limit for these cardiac fat depots [26]. The main techniques include echocardiography, computed tomography (CT), and magnetic resonance imaging (MRI), each with distinct advantages and limitations. Iacobellis *et al*. [27] introduced the measurement of EAT using transthoracic echocardiography (TTE) in 2003, providing a simple, noninvasive, and reliable method for visualizing visceral adipose tissue. TTE effectively measures EAT thickness and correlates with myocardial fat content but cannot accurately assess EAT volume or regional fat distribution [28]. While it is cost-effective, the accuracy of TTE depends on operator skill and patient anatomy, whereby it is essential to carefully distinguish between the EAT and PAT during the TTE measurements. Specifically, PAT appears as a hypoechoic space located anterior to the EAT and parietal pericardium and remains relatively stable without substantial deformation during cardiac cycles; CT and MRI are more effective options for precise volume and thickness measurements. CT offers three-dimensional (3D) imaging with high spatial and temporal resolution, enabling accurate quantification of EAT volume and detailed visualization of the heart and epicardial surface. Moreover, CT allows separate measurements for EAT and PAT without contrast agents [26]. The drawback of CT is its exposure to ionizing radiation, which poses potential health risks, particularly following repeated imaging sessions. Conversely, MRI offers a noninvasive and accurate measurement of both EAT/PAT thickness and volume [29]. MRI is operator-independent, not restricted by acoustic windows, and provides a detailed assessment [30]. While MRI circumvents the radiation risks of CT, it has limitations, including longer imaging and analysis times, lower availability, and higher costs.

## 5. The Role of EAT/PAT in Coronary Artery Disease

Atherosclerosis occurs when immune cells and cholesterol build up in the inner arterial layer, specifically in the subendothelial space. When plaque accumulation affects the arteries that supply blood to the heart, it can lead to CAD. EAT, which is metabolically active and contains numerous proinflammatory cytokines, is believed to be associated with early atherosclerosis and is a predictive marker for future cardiovascular events. The involvement of EAT as a contributor to the complex pathways leading to coronary atherosclerosis was initially proposed in the early 2000s. Patients with mixed or noncalcified plaques presented a notably larger EAT volume than those with calcified or without plaques [31]. An elevated volume of EAT is associated with the occurrence of high-risk coronary artery plaque features [32] and is recognized as an independent predictor for cardiovascular risk and CAD [33]. In apolipoprotein E-deficient (*ApoE*^-⁣/-^) mice that underwent transplantation of perivascular visceral fat or SAT adjacent to the right common carotid artery, perivascular visceral fat led to more severe endothelial dysfunction and accelerated atherosclerosis than did SAT [34]. In the EISNER trial (Early Identification of Subclinical Atherosclerosis by Noninvasive Imaging Research), EAT volume, density, and attenuation had prognostic value in predicting future adverse cardiac events, including myocardial infarction (MI), major adverse cardiovascular events, and cardiac death, in asymptomatic subjects without known CAD [35, 36]. However, findings on the relationship between EAT density and CAD remain inconsistent. For instance, Hell *et al*. [37] reported a notable increase in EAT volume in patients after MI while observing no significant changes in EAT density. On the other hand, Liu *et al*. [38] found EAT attenuation positively associated with CAD risk factors, such as age, BMI, cholesterol levels, neutrophil-to-lymphocyte ratios, and coronary artery calcium score. A meta-analysis suggests that CAD patients generally exhibit higher EAT densities than healthy individuals, with pooled density values averaging –80.71 HU in CAD patients versus –86.40 HU in healthy controls [39]. This inconsistency may stem from several factors impacting EAT density measurements. For example, variations between unenhanced and contrast-enhanced CT scans can yield inconsistent density values, with differences in EAT segmentation methods potentially further influencing results [39].

EAT can contribute to CAD through complex mechanisms, including inflammation, an overactive innate immune response, oxidative stress, damage to the endothelium, stress on adipocytes, the accumulation of lipids, and the effects of high glucose levels. Inflammation is a key characteristic of EAT in patients with CAD, marked by the infiltration of immune cells such as macrophages, mast cells, and CD8+ T cells. Indeed, the balance skews heavily toward proinflammatory M1 macrophages within the EAT, which vastly outnumber their anti-inflammatory M2 counterparts. This inflammatory environment is further supported by the elevated expression of proinflammatory cytokines such as interleukin-6 (IL-6), C-C motif chemokine ligand 2 (CCL2), and tumor necrosis factor-alpha (TNF-α), along with an increase in chemokine ligands, receptors, and proinflammatory adipokines, such as chemerin, resistin, serglycin, and intelectin 1. These changes contribute to the inflammatory profile observed in the EAT of CAD patients [40, 41]. Notably, the level of EAT inflammation is higher than in the SAT and surpasses any other visceral fat depot. Additionally, EAT adipocytes from patients with more severe coronary atherosclerosis presented higher rates of oxidative stress [42]. In individuals with obesity, metabolic syndrome, or CADs, EAT secretes reduced levels of protective adiponectin while increasing the production of harmful leptin, leading to elevated TNF-α, inflammation, and oxidative stress [43]. These elevated leptin levels also promote monocyte adhesion and the transformation of macrophages into foam cells within the arteries, causing detrimental alterations in the EAT and the development and instability of atherosclerotic plaques [44].

EAT acts as a local source of ectopic lipids, contributing to the buildup of lipids in coronary arteries through the overproduction and release of fatty acids by epicardial fat cells that penetrate the adventitia. Notably, group II secretory phospholipase A2 levels, the enzyme that generates proinflammatory lipids, are significantly higher in the EATs of CAD patients than in healthy individuals [45]. Additionally, EAT can promote atherosclerosis through mechanical effects. Under normal conditions, EAT reduces arterial flexibility, whereas excessive amounts of EAT around the coronary arteries may aggravate asymmetric vascular remodeling. Indeed, coronary lesions surrounded by EAT exhibit easier vessel wall expansion due to extravascular resistance than those surrounded by the myocardium [46].

Although the exact mechanism through which EAT is involved in atherosclerosis remains unclear, measuring EAT has been explored in research settings as a potential tool for risk assessment in CAD. Patients with CAD typically exhibit greater EAT volume and thickness than individuals without CAD [37, 47]. While routine clinical risk stratification is not currently recommended in the CAD guidelines, research suggests that EAT volume may be associated with coronary artery calcium score irrespective of body size, body fat, and traditional cardiovascular risk factors in men but not women [48]. Moreover, researchers have identified amending associations with EAT as a strategy to potentially delay the progression of atherosclerosis. Indeed, reducing RPS3A in periaortic adipose tissue in an atherosclerosis mouse model impaired the browning process in perivascular adipose tissue, leading to increased vascular inflammation and accelerated atherosclerosis development [49]. Furthermore, EAT resection effectively slowed the development of atherosclerosis in pig CAD models [50, 51].

## 6. The Role of EAT/PAT in Atrial Fibrillation

Epidemiological and clinical studies have consistently shown strong associations between EAT/PAT and the presence, severity, and recurrence of AF after ablation [52]. In the Framingham Heart Study cohort, PAT volume, but not intrathoracic or visceral abdominal fat volume, was associated with prevalent AF after adjusting for AF risk factors, including BMI, heart failure, myocardial infarction, and intrathoracic fat volume [53]. A recent systematic meta-analysis of such studies compared the strength of associations between EAT and AF and reported that every 1 increase in standard deviation relating to the EAT volume was associated with a 2.2-fold increase in AF risk [54]. In addition to changes in volume, studies have shown that the mean fat attenuation index was significantly higher in AF patients [55], and EAT density is an independent predictor of postoperative AF following simple aortic valve replacement [56]. Notably, while obesity is a known risk factor for AF, the link between PAT/EAT and AF, to some extent, is not reliant on obesity and, in some cases, is completely independent. In the Framingham Heart Study, which involved 2317 participants, PAT volume predicted AF risk independent of other adiposity measures, with an odds ratio (OR) of 1.28 per standard deviation increase in PAT volume, even after adjusting for other AF risk factors [53]. Batal *et al*. [57] evaluated 169 patients and reported that posterior left atrial fat thickness was associated with AF burden independent of left atrial area and BMI, with a 1 cm increase in fat thickness correlated with an OR of 6.06. Similarly, Al Chekakie *et al*. [58] demonstrated that pericardial fat volume predicted AF independently of BMI, alongside traditional risk factors and left atrial enlargement. Wong *et al*. [59] reported that atrial PAT volume predicted AF incidence and severity after adjusting for body weight, with an OR of 5.33 for periarterial fat and 11.97 for periventricular fat.

Multiple mechanisms have been proposed to explain how PAT/EAT leads to AF. These mechanisms can be summarized from two aspects: (1) physical conduction block due to extensive fibrosis and (2) local infiltration of the adjacent atrial myocardium, leading to conduction heterogeneity and electrophysiological alterations through the release of cytokines that disrupt intercardiomyocyte adhesion, affect cell coupling, modify ionic currents, influence myocardial metabolism, trigger inflammation, and amend the electrical or structural characteristics of the atrium. The atrial EAT secretome from AF patients slows conduction, depolarizes the resting potential, alters electrical cell–cell coupling, and facilitates re-entrant arrhythmias in cardiomyocytes [60]. The EAT secretome from AF patients induces extracellular matrix (ECM) gene expression in atrial fibroblasts and contains abundant myeloperoxidase, the latter of which aggregates in the subepicardial and around fibrofatty infiltrates [61]. EAT releases profibrotic factors (activin A, connective tissue growth factor (cTGF), matrix metalloproteinases (MMPs), and transforming growth factor (TGF)β1 and TGFβ2) and microRNAs via secretion or through extracellular vesicles (EVs), inducing atrial myopathy and fibrillation [62, 63]. Immunoblot analysis detected aromatase expression in both human atrial appendage and EAT samples. Meanwhile, in rodent models, aromatase levels in the myocardium and PAT showed more than a 20-fold increase compared to young control animals. When comparing young and aged animals on a high-fat diet, there was a strong positive correlation between the total aromatase levels in PAT and the incidence and duration of triggered AF [64]. In addition to bioactive molecules, EAT is a lipid reservoir that supplies lipids to the adjacent atrium. The transported free fatty acids affect atrial tissue electromechanics, potentially separating cardiomyocytes, leading to reduced conduction speed, disruption of cell connections, and disorganization of the myocardial structure. These effects can contribute to conduction delays and re-entry phenomena [65]. Ganglionated plexi are located in EATs and play a vital role in initiating and sustaining AF. A previous study found that injecting the neurotransmitter inhibitor botulinum toxin into the EAT resulted in suppressed AF inducibility, reduced the incidence of atrial tachyarrhythmia and burden during the 3-year follow-up, and reduced hospitalizations [66, 67]. Subsequently, a recent phase 2 trial, the NOVA study (NCT03779841), examined using botulinum toxin type A (AGN-151607) to prevent postoperative AF in patients undergoing open-chest cardiac surgery. The results revealed no significant differences in postoperative AF rates after administering AGN-151607. However, a subgroup analysis showed that older patients aged 65 years and above who underwent coronary artery bypass grafting experienced lower rates of postoperative AF and rehospitalization [68]. This evidence highlights the complex role of EAT in AF and suggests that targeting EAT could represent a new therapeutic strategy for treating AF.

## 7. The Role of EAT and PAT in Heart Failure

Heart failure often represents a late stage of left ventricular remodeling (LVR), which can involve either cardiac hypertrophy or ventricular dilation. Moreover, depending on the underlying condition, this may lead to either a thickened or thinned left ventricular wall, as observed in conditions such as myocardial infarction or hypertensive heart disease [69]. This process involves several critical steps, including modifications in myocyte biology, the myocardium, and the geometry of the left ventricular chamber. The underlying mechanisms driving LVR likely involve apoptosis, oxidative stress, and the proliferation of fibroblasts. A greater volume of EAT, which is related to increased susceptibility to heart failure, is correlated with atrial enlargement and compromised diastolic filling in both the right and left ventricles [70]. A meta-analysis of 22 studies reported that increased EAT was independently linked to diastolic dysfunction, regardless of other measures of adiposity [71]. Notably, nearly 50% of patients presented with heart failure with preserved ejection fraction (HFpEF, ejection fraction (EF) >40%), and evidence suggests that EAT may contribute to the pathophysiology of HFpEF. Studies indicate that EAT volume is significantly higher in patients of HFpEF than in healthy controls, although only a few account for confounding factors such as CAD or obesity [72, 73, 74, 75, 76]. Accumulation of EAT has been linked to poor outcomes, including all-cause mortality and first heart failure hospitalizations in individuals with heart failure with mid-range ejection fraction and HFpEF [77]. Gorter *et al*. [78] reported that increased EAT was associated with elevated right-sided filling pressures and reduced exercise capacity in patients with HFpEF, independent of pulmonary vascular resistance and obesity. Indeed, in patients with HFpEF, regional atrial EAT was higher in those with AF than those without the condition [79]. Following a comprehensive review, Rossi *et al*. [80] summarized the role of EAT across the heart failure spectrum, particularly for HFpEF. Moreover, Rossi *et al*. [80] highlighted that EAT tends to acquire a proinflammatory profile, with increased EAT volume correlating with poorer clinical outcomes from HFpEF [80]. On the other hand, the current understanding of EAT in heart failure with reduced ejection fraction (HFrEF, EF <40%) is limited. Several studies proposed that the role of EAT in HFrEF contrasts with that in HFpEF, as lower EAT levels are associated with more pronounced left ventricular dysfunction, greater functional impairment, and worse outcomes [76, 81]. The reduced EAT volume in HFrEF patients likely reflects the catabolic state and adverse left ventricular remodeling associated with advanced heart failure. This variation may be influenced by comorbidities such as CAD, obesity, and diabetes, affecting EAT volume in HFrEF. Additionally, the metabolic and hemodynamic status of HFrEF patients plays a role, with those in more severe stages of illness often showing systemic fat loss, including a reduction in EAT volume.

EAT influences cardiac function in heart failure through multiple mechanisms, such as elevated inflammation, increased fibrosis, disruption of autonomic regulation, and the mechanical impact of a large, fibrotic fat mass. Moreover, the proteomic profile of EAT was shown to contribute to the development of heart failure [82, 83]. Compared with SAT, EAT results in elevated levels of adipokines, such as leptin, IL-6, TNF-α, and adiponectin, which play significant roles in paracrine signaling pathways, potentially contributing to myocardial lipotoxic cardiomyopathy and inflammatory responses in the context of heart failure [2, 18].

Mechanically, an increased volume of EAT strongly correlates with the impairment of left ventricular diastolic relaxation and filling, emphasizing the significant role of EAT in cardiac dynamics. Moreover, the lack of fascial separation between EAT and the myocardium facilitates the infiltration of lipids into the myocardium. The consequential assimilation of excessive fatty acids by cardiomyocytes leads to ectopic myocardial lipid accumulation, a phenomenon that contributes significantly to the development of heart failure. This process induces cardiomyocyte disarray, dysfunction, and apoptosis. Furthermore, individuals with HFpEF present a notably greater content of intramyocardial fat than do those with HFrEF or individuals without heart failure [84]. The elevated intramyocardial fat content is strongly associated with markers of left ventricular dysfunction in individuals with HFpEF [76], highlighting the intricate interplay between EAT, myocardial lipid dynamics, and heart failure progression.

With respect to PAT, little attention has been given in clinical research to its relationship with heart failure. A prospective cohort study including 6784 participants reported PAT volume, defined as the sum of EAT and paracardial fat, was strongly associated with an increased risk of HFpEF but not HFrEF [85]. In mice, surgical removal of PAT (80% PAT) before left anterior descending artery ligation inhibited left ventricular fibrosis at 7 days post-MI, suggesting that PAT adversely affects post-MI outcomes [86]. Our group attempted to replicate their PAT removal experiment. However, due to the abundant vascular tissue present in PAT, excessive removal in our study led to significant hemorrhage around the mouse heart, affecting survival rates. Conversely, minimal PAT removal had negligible effects on cardiac function, possibly due to compensatory actions exerted by the remaining PAT (data not published). Consequently, the surgical technique and extent of PAT removal in this study significantly influenced the success rate of modeling. Some PAT-related proteins might be beneficial. For example, exosomes derived from PAT transport adipsin to myocardial tissues, which protects cardiomyocytes against ferroptosis and maintains iron homeostasis after MI [87]. Notably, exosomes were isolated from normal PAT in this study. As a metabolically active visceral fat depot, the secretome of PAT or EAT might be altered in response to disease stimuli, transitioning from a protective role to a destructive role. A proteomic analysis of EAT was performed in heart failure and nonheart failure patients, identifying 771 proteins using liquid chromatography–tandem mass spectrometry. Among these, 17 proteins were found to be more abundant in heart failure patients, while 7 showed reduced levels [82]. The differentially expressed proteins were primarily associated with reactive oxygen species responses, oxidative stress, inflammation/immune responses, and lipid metabolism [82]. These findings underscore the dual role of PAT and EAT, which may shift from protective to harmful depending on the disease context, highlighting the need for a deeper understanding of their distinct contributions to heart failure and other cardiovascular conditions.

## 8. Targeting EAT/PAT in Cardiovascular Diseases

The recognition of EAT and PAT as active players in cardiovascular pathology presents novel opportunities for therapeutic intervention. Evidence suggests that a lower EAT volume coupled with higher EAT density is associated with improved ejection fraction in heart failure patients (HFimpEF, defined previously as HFrEF patients with an absolute left ventricular ejection fraction (LVEF) improvement ≥10% and a second LVEF >40% after at least 3 months) [88]. This insight points toward a dual approach: reducing EAT/PAT quantity and enhancing quality through anti-inflammatory or metabolic-targeting interventions.

### 8.1 Non-Pharmacological Interventions

Lifestyle changes remain the cornerstone of CVD prevention and management. Weight loss achieved through caloric restriction and increased physical activity has been shown to reduce EAT thickness [89]. A 12-week aerobic exercise program significantly reduces EAT in overweight and mildly obese individuals [90]. Adherence to heart-healthy diets, such as the Mediterranean diet, rich in anti-inflammatory nutrients, is associated with a significantly lower amount of EAT in patients with AF [91]. In addition to lifestyle modifications, bariatric surgery offers another non-pharmacological approach to reducing adipose tissue depots. Gaborit *et al*. [92] reported that while bariatric surgery significantly reduces overall body weight and visceral adipose tissue (VAT), the reduction in EAT is relatively modest and less pronounced compared to VAT loss. Moreover, myocardial triglyceride content remained unchanged post-surgery, suggesting a limited effect of bariatric surgery on cardiac ectopic fat depots [92]. Conversely, a more recent study reported that although EAT reduction following bariatric surgery is less significant than VAT loss, this modest decrease in EAT has meaningful implications for cardiac geometry and function [93]. EAT loss correlates with decreased pericardial restraint, as evidenced by changes in the left ventricular eccentricity index, leading to improvements in cardiac chamber expansion and function over time [93]. These findings underscore the need for targeted interventions to reduce EAT effectively and address its associated cardiovascular risks.

### 8.2 Pharmacological Interventions

In addition to non-pharmacological strategies, pharmacological interventions have been investigated for their potential to target EAT and PAT, offering additional avenues for cardiovascular disease management. Existing studies have explored the potential therapeutic effects of glucose- and lipid-lowering drugs in reducing EAT. Among them, the glucose-lowering drugs glucagon-like peptide-1 receptor (GLP-1R) agonists and sodium-glucose co-transporter-2 (SGLT2) inhibitors were proposed to exert beneficial cardiovascular effects and reduce EAT. A study of 17 CAD patients undergoing coronary artery bypass grafting revealed that GLP-1R expression in EAT directly correlates with genes promoting beta-oxidation and white-to-brown adipocyte differentiation and is inversely correlated with pro-adipogenic genes. Additionally, GLP-2R expression was positively correlated with genes involved in adipogenesis and lipid synthesis and negatively correlated with genes promoting beta-oxidation. Circulating levels of GLP-1 and GLP-2 are elevated in CAD patients, particularly those with increased EAT thickness, indicating potential cardiovascular implications of these hormones [94]. GLP-1 analogs may target GLP-1R in EAT, reducing local adipogenesis and inducing brown fat differentiation. The GLP1R agonists used in treating type 2 diabetes and obesity provide cardiovascular benefits beyond glucose control, including a reduction in EAT thickness and the modulation of metabolic pathways [95, 96]. Notably, GLP1R is expressed in EAT but not SAT, supporting the hypothesis of a direct effect on this fat depot [97]. The activation of GLP-1/GLP-1R in EAT can lead to reduced local adipogenesis, improved fat utilization, and the induction of brown fat differentiation, potentially contributing to overall cardiovascular benefits. Importantly, the presence of GLP1R in human cardiomyocytes adds an intriguing dimension to the potential cardiovascular impact of these medications. However, elevated circulating GLP-1 and GLP-2 and increased GLP-2R in EAT pose questions about the compensatory mechanisms involved in CAD and EAT expansion and require further exploration in future investigations.

Selective SGLT2 inhibitors, initially designed as oral antidiabetic agents, have emerged as promising treatments for HFpEF and HFrEF, regardless of diabetes status. Clinical trials have demonstrated their efficacy in reducing major adverse cardiovascular events, cardiovascular death, and heart failure. Notably, SGLT2 inhibitors, such as dapagliflozin and empagliflozin, significantly reduce EAT thickness or volume, independent of weight loss [98, 99, 100]. Through glycosuria-induced shifts in substrate utilization, SGLT2 inhibitor therapy promotes increased fatty acid oxidation, lipolysis, and ketogenesis and improves myocardial glucose metabolism [101]. While the cardiovascular benefits of EAT lipolysis resulting from SGLT2 inhibitor therapy have yet to be conclusively demonstrated, potential mechanisms can be postulated based on available data. A recent study revealed that SGLT2 is primarily expressed in human preadipocytes within EAT, and its expression significantly declines as preadipocytes undergo terminal differentiation. Empagliflozin, an SGLT2 inhibitor, effectively inhibits the differentiation and maturation of human epicardial preadipocytes and enhances the paracrine secretory profile of EAT, particularly by regulating IL-6 expression [102]. Another SGLT2 inhibitor, dapagliflozin, boosts glucose uptake in EAT, reduces the release of proinflammatory chemokines, and improves the differentiation process of EAT cells [103]. A recent study further demonstrated that SGLT2 inhibitors provide benefits beyond EAT, reducing interstitial myocardial fibrosis, aortic stiffness, and inflammation markers in non-diabetic patients with HFrEF [98]. The SGLT2 inhibitors decrease EAT, improve metabolic pathways and enhance both systolic and diastolic functions, underscoring the need for further research to clarify their specific effects on EAT.

In addition to glucose-lowering medications, statins, a class of lipid-lowering drugs, have been reported in several studies to reduce EAT thickness [104, 105]. However, the observed effect is less prominent than that of GLP1R agonists and SGLT2 inhibitors [106]. Statins may reduce EAT by modulating peroxisome proliferator-activated receptors (PPARs), particularly through the activation of PPARα and PPARγ, which is associated with improved insulin sensitivity and increased glucose uptake in EAT [105]. Interestingly, a novel class of lipid-lowering drugs, proprotein convertase subtilisin/kexin 9 (PCSK9) inhibitors, has demonstrated a more substantial reduction in EAT after a 6-month intake (~20%), independent of low-density lipoprotein cholesterol (LDL-C) levels [107]. These findings highlight the efficacy of lipid-lowering drugs in reducing EAT and mitigating inflammation. However, a comprehensive understanding of the underlying mechanisms governing the effects of lipid-lowering medications on EAT requires further exploration.

Transcriptional analysis revealed that EAT displays elevated expression of the beige adipocyte-specific marker CD137, as well as the thermogenic genes *UCP-1*, *PRDM16*, *PGC-1α*, and *PPARγ*, and beige adipose tissue (BAT)-specific genes, such as *ACTA1*, *PPARGC1A*, *troponin C type 1*, and *troponin I type 1*, in comparison with SAT, resulting in a beige/BAT-like appearance [108, 109]. However, the thermogenesis ability of EAT may decrease with age, obesity, and CAD [110]. Notably, the beiging or browning of EAT could offer cardiovascular advantages. Adipose-derived EVs represent a new mechanism of interorgan and intercellular communication between EAT and the cardiovascular system, playing a role in promoting the browning of white adipose tissue (WAT). A study has shown a one-way transfer of exosomes from adipose tissue-derived stem cells (ADSCs) to macrophages, driving their polarization toward the anti-inflammatory M2 subtype. Additionally, these ADSC-derived exosomes were found to promote WAT browning in mice inguinal and epididymal regions [111]. Researchers are investigating the components of ADSC EVs that induce the browning of WAT. miR-196a has been demonstrated to induce WAT browning under cold exposure conditions and β-adrenergic stimulation [112]. Additionally, miR-155 has been found to enhance brown adipose tissue function and cause a brown adipocyte-like phenotype in white adipocytes [113]. However, further research is necessary to elucidate the specific role of EVs and their yet-to-be-fully-understood content in the formation of beige adipocytes and the transition from white to beige adipogenesis.

Notably, the browning/beiging process involves regulating specific genes, signaling pathways, and crucial aspects of mitochondrial dynamics. Increasing the number of mitochondria and enhancing mitochondrial biogenesis are integral steps in this process, ensuring that beige adipocytes effectively fulfill their thermogenic role. In CAD patients, mitochondrial respiratory capacities, including oxidative phosphorylation (OXPHOS) with both nonfatty acid substrates (linked to complex I and complex I + II) and fatty acids, are markedly reduced in EAT [114]. Recently, we reported that myocardial injection of exogenous stem cell-derived mitochondria has a protective effect on postinfarction cardiac function [115]. Hence, investigating the potential of exogenous mitochondrial supplementation in preserving the quantity and function of mitochondria in EAT/PAT, thereby maintaining beige fat characteristics and ultimately supporting cardiac functional homeostasis in diseased environments, represents a promising research avenue.

## 9. Future Perspective

**Fig. 2.**
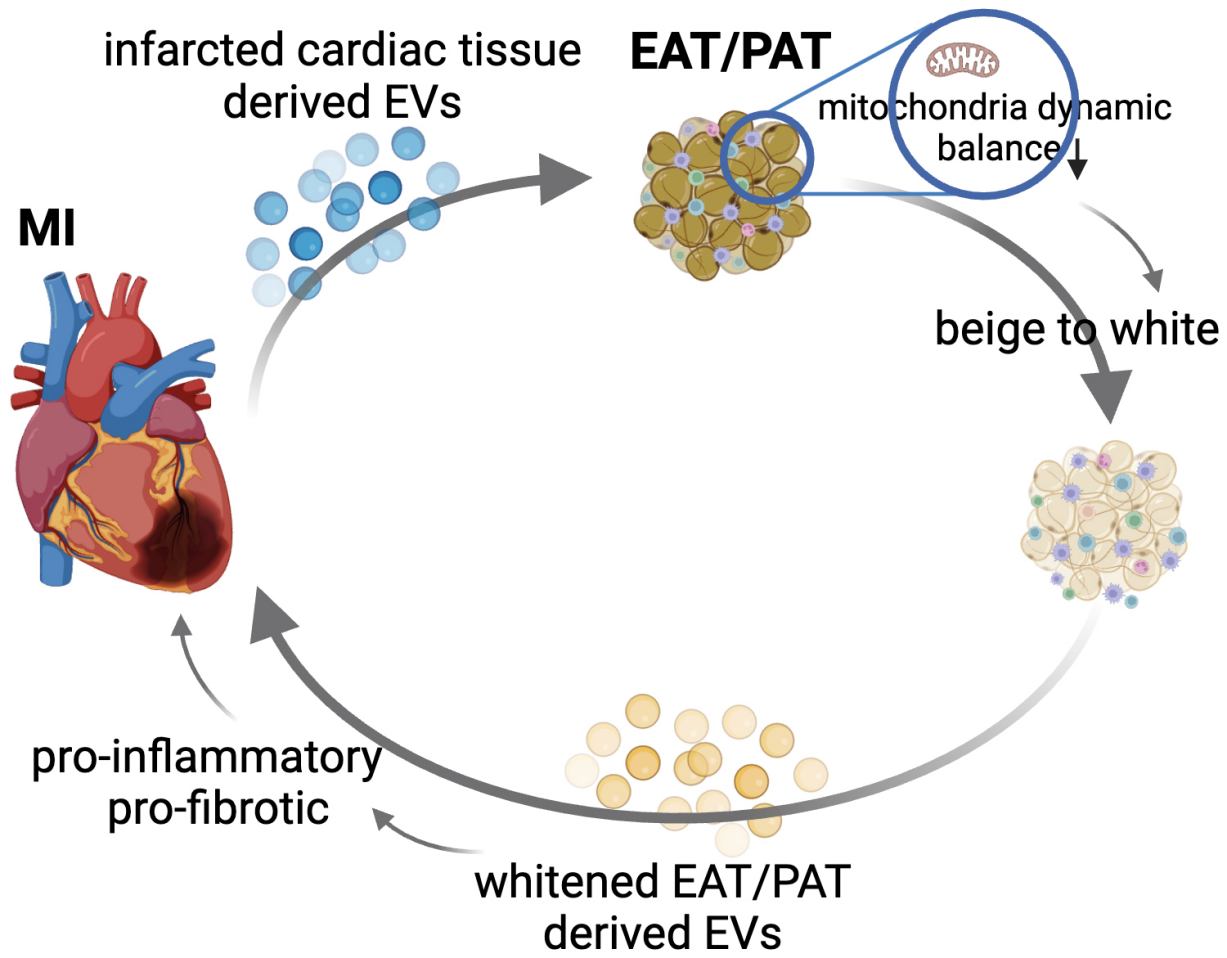
**Proposed bidirectional signaling between cardiac tissue and EAT/PAT via extracellular vesicles (EVs) in the progression of cardiovascular disease (CVD)**. This figure illustrates the hypothesized reciprocal relationship between diseased cardiac tissue and EAT or PAT via the release of tissue-derived EVs. Under disease conditions such as myocardial infarction (MI) or atrial fibrillation (AF), cardiac tissue releases proinflammatory and profibrotic EVs. These EVs may target adjacent fat depots such as EAT or PAT, driving pathological beige-to-white processes by disrupting the balance in mitochondrial dynamics. In response, the diseased EAT/PAT secrete harmful EVs that further exacerbate inflammation and fibrosis in the heart, creating a vicious cycle of mutual deterioration. Thus, disrupting this signaling loop could provide novel therapeutic targets for preventing or reversing cardiovascular disease progression. EAT/PAT, epicardial adipose tissue/pericardial adipose tissue.

Exosomes or EVs have emerged as powerful mediators of cell-to-cell communication, influencing various biological processes, including tissue repair, inflammation, and immune modulation. These vesicles contain bioactive molecules such as proteins, lipids, and RNAs (e.g., microRNAs), which can influence the behavior of adjacent or even distant cells or tissues. Our group reported that myocardial ischemia-reperfusion (IR) significantly increases the release of cardiac EVs, which have proinflammatory properties and exacerbate heart injury, while IR injury was reduced by inhibiting EV release using GW4869 [116]. Shaihov-Teper *et al*. [62] demonstrated that EVs from EAT (EAT EVs) from patients with AF carried a distinct proinflammatory, profibrotic, and pro-arrhythmic profile compared to non-AF patients. These EAT EVs enhanced fibrosis and inflammation in the atrial myocardium and induced arrhythmic re-entry in cardiomyocytes, suggesting a novel mechanism through which EAT contributes to AF pathogenesis [62]. While research on EVs from EAT or PAT remains limited, current findings allow us to propose a compelling hypothesis: Under disease conditions, cardiac tissue-derived EVs influence adjacent fat depots, such as EAT or PAT, potentially promoting their whitening. This transformation could lead to the secretion of EVs by diseased EAT or PAT, which no longer supports cardiac tissue repair but instead aggravates inflammation and fibrosis. This reciprocal influence could create a vicious cycle, where worsening cardiac function exacerbates fat depot pathology and vice versa (Fig. 2). Thus, exploring the bidirectional signaling between cardiac tissue and EAT/PAT via EVs may help disrupt the pathological cycle, potentially offering a targeted intervention to halt or reverse the progression of heart disease. For instance, as discussed earlier, the mitochondrial dysfunction observed in EAT/PAT after cardiac injury could play a pivotal role in the beige-to-white process. This process may reduce the beneficial thermogenic properties of EAT/PAT and promote the release of harmful EVs that further exacerbate myocardial injury. From this aspect, supplementing exogenous mitochondria to EAT/PAT could be an innovative therapeutic approach to restore mitochondrial dynamics and maintain their beige fat characteristics. Improving mitochondrial health may inhibit the pathological secretion of proinflammatory and profibrotic EVs from EAT/PAT, thereby reducing their detrimental effects on the diseased myocardium.

## 10. Conclusions

The primary objective of this review was to delineate the respective roles of EAT and PAT in CVD. However, during literature retrieval, a significant number of clinical reports were found to exist with respect to the association between EAT and CVD, owing to its close anatomical proximity and vascular supply to the myocardium. Conversely, a notable lack of clinical research focuses on the association between PAT and CVD. The lack of appropriate animal models, particularly the absence of EAT in rodent models, has hindered a comprehensive understanding of the mechanisms through which EAT participates in structural and functional cardiac and coronary abnormalities. Consequently, in basic research, PAT is often reluctantly utilized as the tissue closest to EAT. Nevertheless, despite their adjacency to the heart, EAT and PAT may exhibit distinct effects and mechanisms of action. Some scholars have undertaken comparisons between them. Sissel Åkra and colleagues [117] investigated the gene expression and protein secretion related to the nucleotide-binding oligomerization domain (NOD)-like receptor protein 3 (NLRP3) inflammasome inflammatory pathway in EAT, PAT, and SAT from coronary heart disease (CHD) patients undergoing open-heart surgery. In non-obese CHD patients, EAT exhibited elevated levels of interleukin-18 (IL-18) and IL-6, while PAT demonstrated NLRP3 inflammasome activation comparable to SAT [117]. Sissel Åkra and colleagues [117] further examined the expression of the senescence marker sirtuin 1 (SIRT1) and nicotinamide phosphoribosyltransferas (NAMPT) enzyme, which regulates SIRT1 activity, in EAT, PAT, and SAT from CHD patients, using individuals with aortic valve disease as controls. No significant differences were found in the SIRT1 and NAMPT expressions in adipose tissues or circulating levels between CHD patients and controls, possibly due to the proinflammatory state in the control group with aortic valve disease [117]. However, the expression levels of SIRT1 and NAMPT among CHD patients differed across tissue types, with SIRT1 expression in PAT and SAT found to be higher than in the EAT. Further analysis suggested that SIRT1 has an anti-inflammatory function; therefore, the SIRT1 profile in CHD patients might have been adversely affected by prolonged chronic inflammation, with EAT showing the highest level of inflammation compared with PAT and SAT [118]. These findings underscore the distinct roles and responses of EAT and PAT in cardiovascular diseases. Further exploration of their unique mechanisms may provide valuable insights into targeted therapeutic interventions for cardiovascular disorders.
